# Cell-Permeable Parkin Proteins Suppress Parkinson Disease-Associated Phenotypes in Cultured Cells and Animals

**DOI:** 10.1371/journal.pone.0102517

**Published:** 2014-07-14

**Authors:** Tam Duong, Jaetaek Kim, H. Earl Ruley, Daewoong Jo

**Affiliations:** 1 Department of Biomedical Sciences, Chonnam National University Medical School, Kwangju, Korea; 2 Division of Endocrinology and Metabolism, Department of Internal Medicine, College of Medicine, Chung-Ang University, Seoul, Korea; 3 Department of Pathology, Microbiology & Immunology, Vanderbilt University School of Medicine, Nashville, Tennessee, United States of America; 4 ProCell R&D Institute, ProCell Therapeutics, Inc., Seoul, Korea; Virginia Commonwealth University, United States of America

## Abstract

Parkinson’s disease (PD) is a neurodegenerative disorder of complex etiology characterized by the selective loss of dopaminergic neurons, particularly in the substantia nigra. Parkin, a tightly regulated E3 ubiquitin ligase, promotes the survival of dopaminergic neurons in both PD and Parkinsonian syndromes induced by acute exposures to neurotoxic agents. The present study assessed the potential of cell-permeable parkin (CP-Parkin) as a neuroprotective agent. Cellular uptake and tissue penetration of recombinant, enzymatically active parkin was markedly enhanced by the addition of a hydrophobic macromolecule transduction domain (MTD). The resulting CP-Parkin proteins (HPM_13_ and PM_10_) suppressed dopaminergic neuronal toxicity in cells and mice exposed to 6-hydroxydopamine (6-OHDH) and 1-methyl-4-phenyl-1,2,3,6-tetrahydropyridine (MPTP). These included enhanced survival and dopamine expression in cultured CATH.a and SH-SY5Y neuronal cells; and protection against MPTP-induced damage in mice, notably preservation of tyrosine hydroxylase-positive cells with enhanced dopamine expression in the striatum and midbrain, and preservation of gross motor function. These results demonstrate that CP-Parkin proteins can compensate for intrinsic limitations in the parkin response and provide a therapeutic strategy to augment parkin activity in vivo.

## Introduction

Parkinson’s disease (PD) is a neurodegenerative disorder characterized by the loss of dopaminergic neurons in the substantia nigra. These striking clinical features have focused efforts to understand the mechanisms responsible for neuronal death and reasons why dopaminergic neurons are differentially affected. An extensive literature implicates oxidative stress, mitochondrial dysfunction and protein misfolding in disease etiology [1], [2], as illustrated by loss-of-function mutations in genes such as *parkin* (*PARK2*), *PINK1* (*PARK6*) and *DJ-1* (*PARK7*) and by the action of toxic agents that induce Parkinson-like diseases in both animals and man.

The parkin protein, functions as an E3 ubiquitin ligase and catalyzes K48 and K62 linked mono- and poly-ubiquitinations involved in protein turnover and trafficking [3]. Parkin substrates include proteins known to accumulate in the neurons of parkin knockout mice; although K62-ubiquitination suggests parkin functions extend beyond protein degradation. The PTEN induced kinase 1 (PINK1) is activated by mitochondrial depolarization and influences parkin recruitment to distressed mitochondria and their subsequent removal by mitophagy. DJ-1, although associated with diverse functions, appears to play a parallel protective role to that of parkin/PINK1 in oxidative stress response. Agents capable of inducing stable Parkinson-like symptoms include chemical neurotoxins, notably 1-methyl-4-phenyl- 1,2,3,6-tetrahydropyridine (MPTP), rotenone and 6-hydroxy-dopamine (6-OHDA) and α-synuclein, a protein that accumulates in Lewy bodies, a clinical signature of human PD [4]. These agents promote neuronal degeneration/dysfunction through a combination of oxidative stress and mitochondrial respiratory impairment.

Despite the complexity of PD etiology, parkin appears to play a broadly protective role in maintaining neuronal function and viability. These protective effects extend to a variety of neurotoxins, mitochondrial poisons and misfolded proteins including: dopamine [5], rotenone and carbonyl cyanide 3-chlorophenylhydrazone [6], 1-methyl-4-phenyl-1,2,3,6-tetrahydropyridine (MPTP), excitotoxin (kainic acid) [7], unfolded protein stress response [8], β-amyloid precursor protein [6], Pael receptor [9], [10], proteasome inhibitors and α-synuclein [11], [12]. Enforced parkin expression also suppresses pathological consequences of PINK1 and DJ-1 gene deficiencies. PINK1 appears to act upstream of parkin, since PINK1 does not complement parkin deficiency. However, both parkin and PINK1 rescue a fragmented mitochondria phenotype of DJ-1 knockout cells, suggesting PINK1/parkin act in parallel with DJ-1 to maintain mitochondrial integrity [1].

These broad cytoprotective activities illustrate the benefits of genetically augmenting parkin levels, and suggest methods to enhance parkin expression and/or activity could provide useful therapies in the treatment of PD. Unfortunately, gene therapy is not a practical option. Moreover, it is not clear if the benefits associated with higher steady-state levels of parkin expression can also be achieved under transient, non-steady state conditions. To address these issues, we developed cell-permeable parkin proteins that we then tested for cytoprotective activity in cultured neuronal cells and in an acute mouse model of PD.

## Results

### Development of Cell-Permeable Parkin Proteins

Multiple hydrophobic macromolecule transduction domains (MTDs) have been used to enhance the delivery of protein cargoes to mammalian cells and tissues [13]–[22]. Similarly, MTD01, MTD10, MTD13, MTD151, and MTD174 were found to enhance the uptake of a His-tagged enhanced green fluorescent protein (EGFP) in RAW cells as assessed by flow cytometry (**Figure S1A**). Relative levels of protein uptake were 0.8 to 3.6 times that of a protein (HM_m_E) containing the membrane translocation sequence (MTS) from FGF4 (Hawiger, 1999) (**Table S1**). By contrast, only minimal levels of protein uptake were observed with a protein (HSP) with an arbitrary peptide sequence in place of the MTD. Similar results were obtained in NIH3T3 cells, using fluorescent microscopy to monitor protein uptake (**Figure S1B**). MTD sequences also enhanced protein delivery to a variety of murine tissues after IP administration (**Figure S1C**).

**Figure 1:**
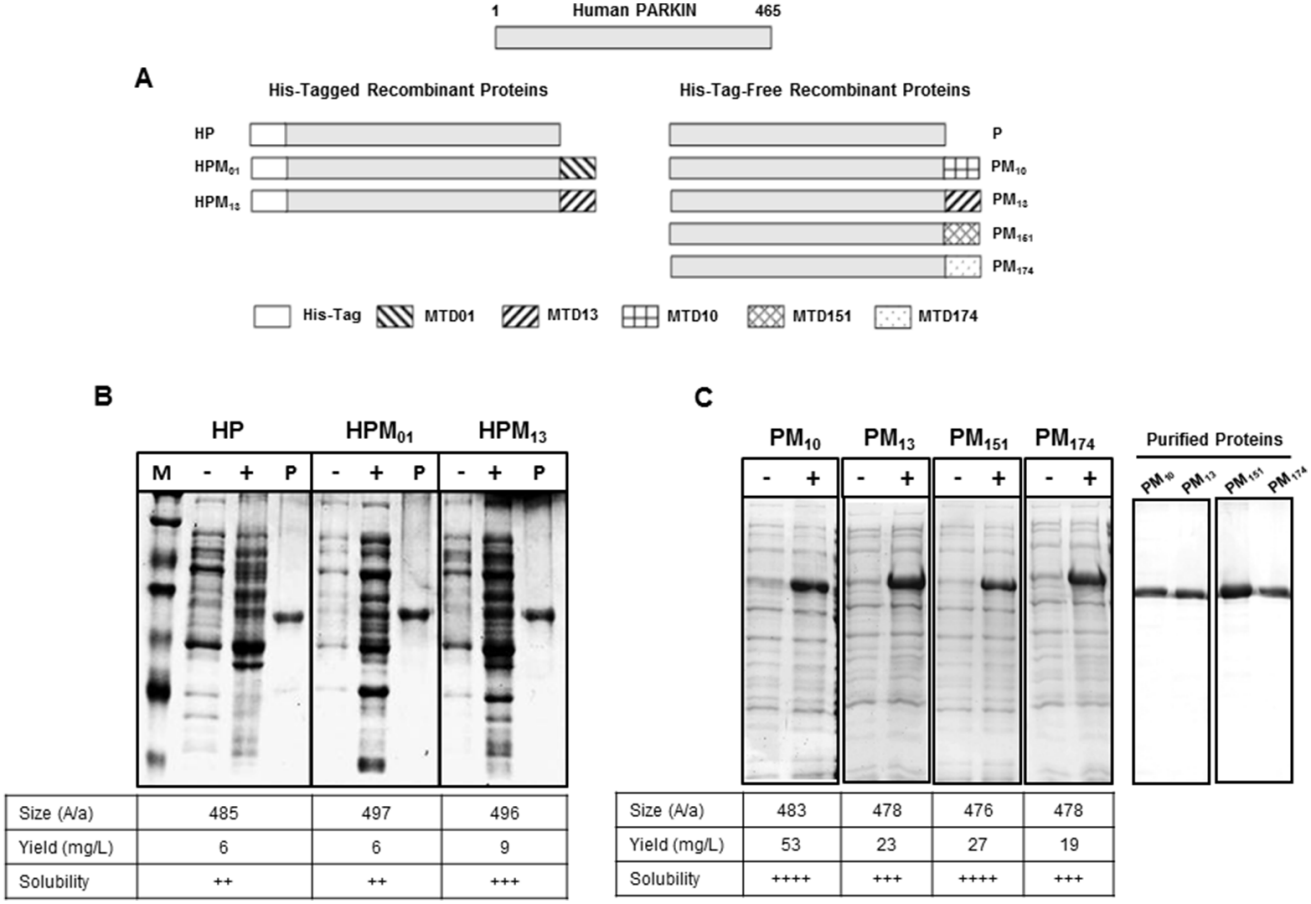
Structure and expression of MTD-parkin fusion proteins. (**A**) Structures of parkin fusion proteins. 6xHis-tagged parkin proteins (left) contained His-tag (HP) only or together with MTD01 (HPM_01_) or MTD13 (HPM_13_) sequences. Parkin proteins without the 6xHis tag (right) included native parkin (P) and proteins containing C-terminal MTD10 (PM_10_), MTD13 (PM_13_) MTD151 (PM_151_) and MTD174 (PM_174_) sequences. (**B–C**) Protein expression in *E. coli*. SDS PAGE analysis of cell lysates before (−) and after (+) IPTG induction; aliquots of Ni^2+^ affinity purified proteins (P); and molecular weight standards (M). The size (number of amino acids), yield (mg/L) and solubility of each recombinant protein are indicated. Solubility was scored on a 4-point scale from highly soluble, with little tendency to precipitate (++++), to largely insoluble proteins (+).

**Figure 2:**
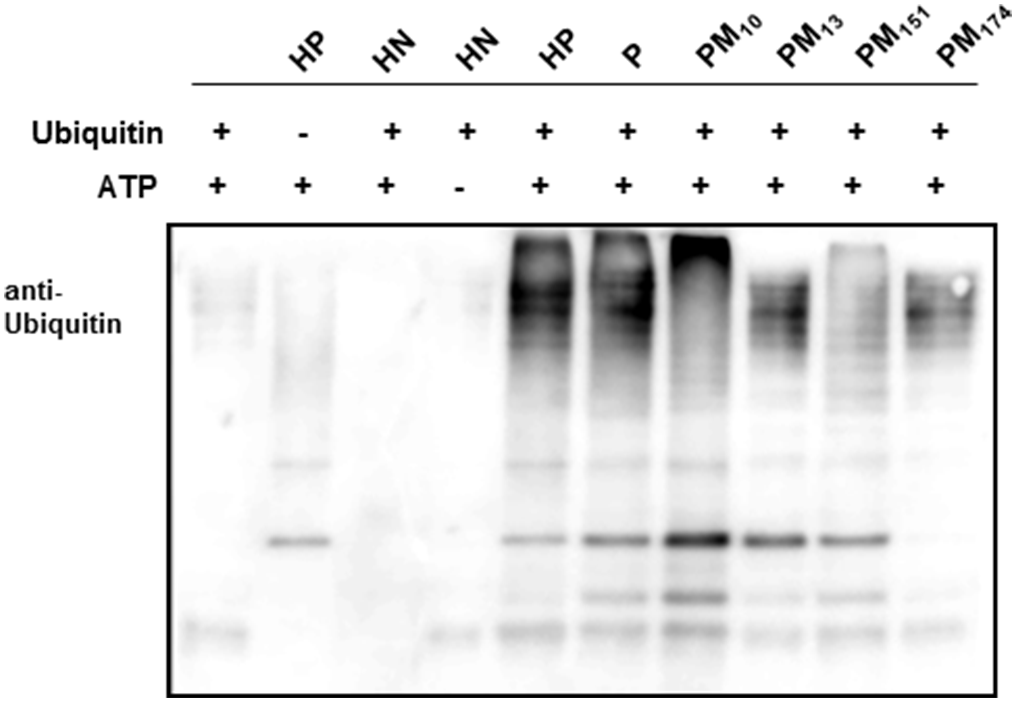
Auto-ubiquitination activity of recombinant parkin proteins. E3 ligase activity of purified recombinant parkin proteins was assessed by an auto-ubiquitination assay. 1 µg of each of the indicated proteins was incubated for 1 hr at 37°C with 1 µM E1, 50 µM E2, 1 mM histidine-tagged Ubiquitin and 10 mM Mg-ATP, and the reaction products were fractionated by SDS PAGE and immunoblotted against an anti-Ubiquitin antibody. Parkin proteins (P, HP, PM_10_, PM_13_, PM_151_ and PM_174_) are described in Figure 1. Samples without individual components or containing an unrelated protein, 6xHis-NM23 (HN), were used as negative controls.

MTD01, MTD10, MTD13, MTD151, MTD174, were subsequently tested for the ability to enhance parkin uptake. Recombinant parkin proteins containing a 6xHis-tag alone (HP) or together with MTD01 and MTD13, were expressed in *E. coli* (**Figures 1A, left panel and 1B**). However, as we were not satisfied with protein solubility and/or yield, additional parkin proteins were expressed that contained no MTD or MTDs 10, 13, 151 or 174 without a 6xHis-tag (designated P, PM_10_, PM_13_, PM_151_, and PM_174_, respectively). In addition, the *PARK2* sequence in these vectors was modified to employ *E. coli* codon preferences (**Figures 1A, right panel and 1C**). The 6xHis-tagged proteins were purified under denaturing conditions by Ni^2+^-affinity chromatography and allowed to refold; whereas the untagged proteins were extracted from inclusion bodies and purified by Q-sepharose anion exchange chromatography (**Figure 1C, right panel**). In each case, proteins lacking the 6xHis tag were expressed at higher levels from codon-optimized vectors and produced greater yields of soluble proteins as compared to 6xHis tagged proteins encoded by the human *PARK2* sequence (**Figures 1B and C** **; bottom panels**). All of the recombinant parkin proteins possessed E3 ubiquitin ligase activity as assessed by using an auto-ubiquitination assay (**Figure 2** and data not shown).

**Figure 3:**
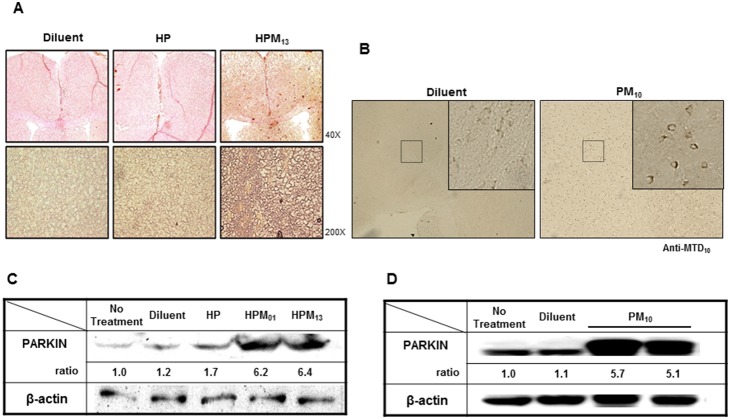
MTD-mediated parkin delivery to the brain. (**A–B**) Immunoblotting of parkin proteins in the cerebellum. Sagittal sections through the cerebellum were immunostatined with anti-parkin (**A**) or anti-MTD10 (**B**) antibody 2 hrs after IP injection of 200 µg of diluent alone or His-tagged parkin proteins without (HP) or with the MTD13 or MTD10 sequences (HPM_13_ or PM_10_) (**C–D**) Western blot analysis of brain parkin. Lysates were prepared from brain samples 2 hrs (**C**) and 30 hrs (**D**) after IV administration of diluent alone or 200 µg His-tagged parkin proteins without (HP) or with the MTD01 (HPM_01_) or MTD13 (HPM_13_) sequences (**C**) or with untagged parkin protein containing MTD10 (PM_10_) (**D**) and analyzed by western blotting using anti-parkin and anti-β-actin antibodies.

Purified parkin proteins were labeled with FITC, and protein uptake was tested either in RAW and NIH3T3 cells by flow cytometry (**Figure S2A**) and fluorescent confocal microscopy (**Figure S2B**), respectively. Since recombinant parkin proteins were positive charged and sticky, it was hard to remove completely the cell surface-bound proteins, resulting in difficulty to distinguish the internalized quantity from surface-bound proteins. We also monitored systemic delivery of CP-Parkin proteins (after IP administration) in a variety of murine tissues (**Figure S1C**). By contrast, 6xHis-tagged parkin without an MTD sequence (HP) did not accumulate in any of the cells or tissues examined. Brain sections and lysates also contained up to 6-fold higher levels of HPM_01_, HPM_13_ and PM_10_ as compared to levels of HP or endogenous parkin proteins as assessed by immunohistochemical staining (**Figures 3A and B**) or Western blot analysis (**Figures 3C and D**). These experiments established MTD01, MTD10 and MTD13 as vehicles for parkin protein delivery both in cultured cells and in animal tissues.

### CP-Parkin Protects Cultured Neuronal Cells from 6-Hydroxydopamine-Induced Apoptosis and Stimulates Dopamine Synthesis

**Figure 4:**
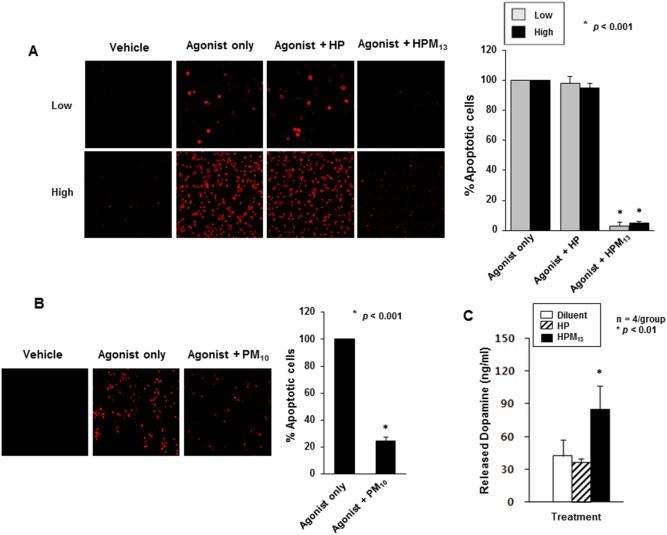
CP-Parkin protects neuronal cells from 6-OHDA-induced apoptosis. (**A**) Suppression of apoptosis in dopaminergic CATH.a cells. CATH.a cells at 5% (Low) or 70% (High) confluence were incubated with 50 µM 6-hydroxydopamine (6-OHDA, Agonist) for 1 hr, treated for 2.5 hrs with 2.5 µM HP or HPM_13_ and assessed for apoptosis by TUNEL staining. The micrographs (left panels) are representative of three independent experiments, plotted (right panels) as means ± S.D. Experimental differences between groups were assessed by a Student’s two-paired *t*-test (*p<0.001). (**B**) Suppression of apoptosis in SH-SY5Y cells. Apoptosis in SH-SY5Y treated with 6-OHDA with and without PM_10_ was assessed as described in (**A**). (**C**) HPM_13_ enhances dopamine release from CATH.a cells. The cells were incubated with 80 µM tyrosine for 24 hrs, treated for 5 hrs with 2.5 µM HP or HPM_13_, and levels of secreted dopamine were measured by ELISA. The data are presented as means ± S.D. of 4 independent experiments. Experimental differences between groups were assessed by a Student’s two-paired *t*-test (*p<0.01 and **p 0.05).

Neuroprotective activities of cell-permeable parkin were tested using an in vitro model of dopamine-induced cytotoxicity. Mouse dopaminergic neuronal cell CATH.a (a catecholaminergic cell line of CNS origin) and SH-SY5Y (derived from a human brain tumor) undergo apoptosis upon treatment with dopamine or dopamine metabolites including 6-hydroxydopamine [23]. As shown in Figure 4A, treatment of CATH.a cells for 1 hr with 50 µM 6-hydroxydopamine (6-OHDA) induced nearly 100% of the cells to undergo apoptosis as assessed by a fluorescent terminal dUTP nick-end labeling (TUNEL) assay. The apoptosis was almost completely blocked by treating cells for 2.5 hrs (starting after 6-OHDA removal) with 2.5 µM HPM_13_ (**Figure 4A**; *p*<0.001). By contrast, HP, a 6xHis tagged parkin protein that lacks an MTD sequence and did not enter cells, was not cytoprotective. Similar results were obtained in SH-SY5Y cells except the cells were exposed to 100 µM 6-OHDA for 6 hrs followed by 2.5 µM PM_10,_ a parkin protein with a different MTD sequence and lacking the 6xHis tag for 2.5 hrs (**Figure 4B**; *p*<0.001).

We also examined the ability of parkin proteins to enhance cellular dopamine release, a marker of normal neuronal function in CATH.a cells, pre-treated with 80 µM tyrosine for 24 hrs. Cells treated with cell-permeable parkin protein (HPM_13_) expressed 2 to 4.8 times higher levels of dopamine 5 hrs after treatment than cells treated with media alone or with 6xHis tagged parkin protein without the MTD sequence (**Figure 4C**; *p*<0.01).

### CP-Parkin Protects Dopaminergic Neurons against MPTP-Induced Neurotoxicity

**Figure 5:**
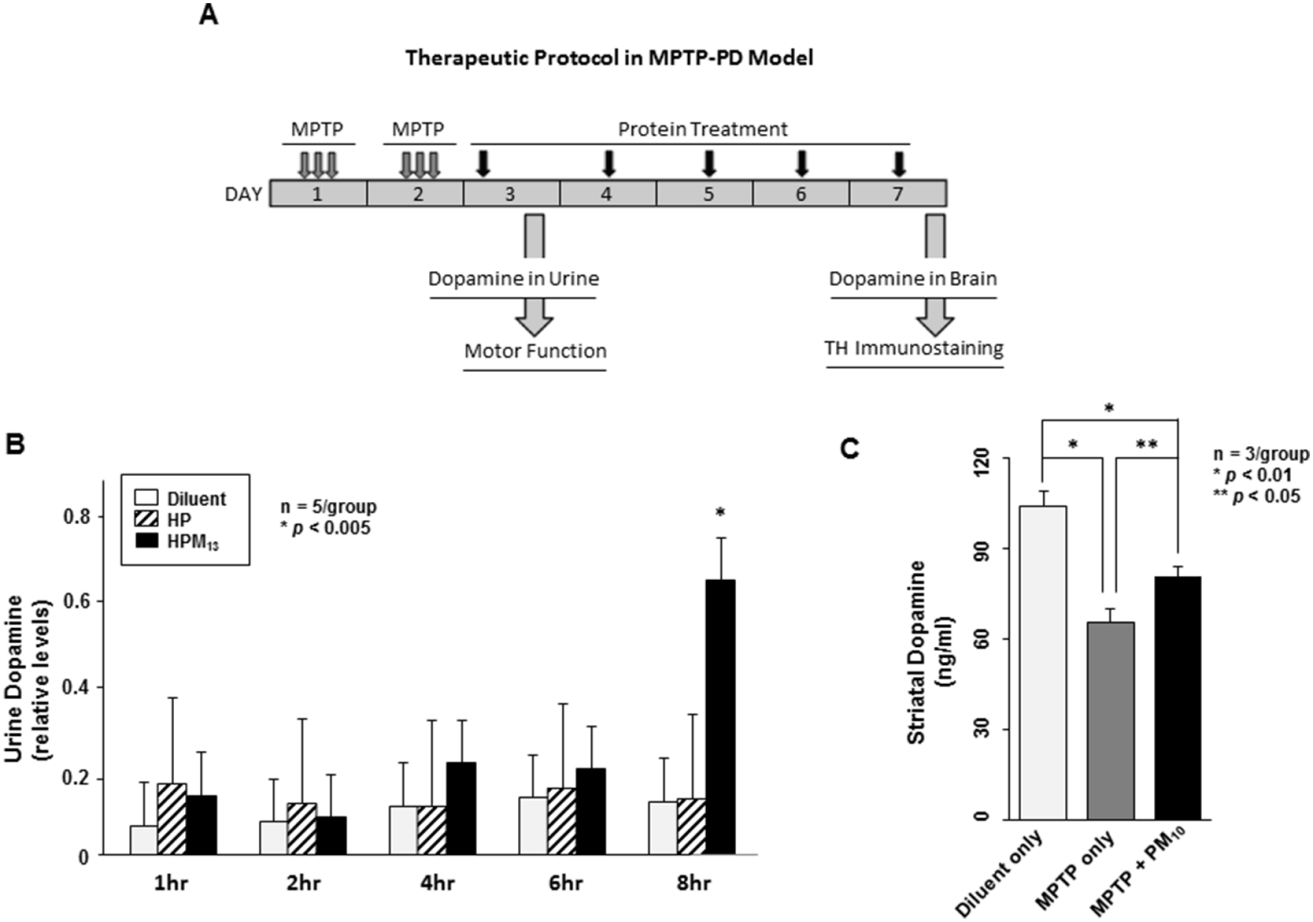
CP-Parkin stimulates dopamine expression in MPTP-lesioned mice. (**A**) Experimental design. 8-week-old C57BL/6 female mice received three doses of MPTP on days 1 and 2 and were injected IP on days 3 through 7 with diluent alone, or with 10 mg/kg of parkin proteins (IP) with (HPM_13_ and PM_10_) or without (HP) a MTD sequence. Urine and brain dopamine levels, gross motor function and brain lesions (TH immunostaining) were analyzed on subsequent days as indicated. (**B**) Dopamine levels in the urine of MPTP-lesioned mice. Urine dopamine levels in MPTP-lesioned mice were measured by ELISA 1, 2, 4, 6 and 8 hrs after HP and HPM_13_ protein treatment. Values from 5 mice are presented as means ± S.D. Experimental differences between groups were assessed by a Student’s two-paired *t*-test (*p<0.005). (**C**) Striatal dopamine levels in MPTP-lesioned mice. Dopamine levels in striatal biopsies were determined by ELISA in lesioned mice without protein treatment or after daily treatments with PM_10_ as shown in panel A. Dopamine levels in groups of 5 mice are presented as means ± S.D. Experimental differences between groups were assessed by a Student’s two-paired *t*-test (*p<0.01 and **p<0.05).

**Figure 6:**
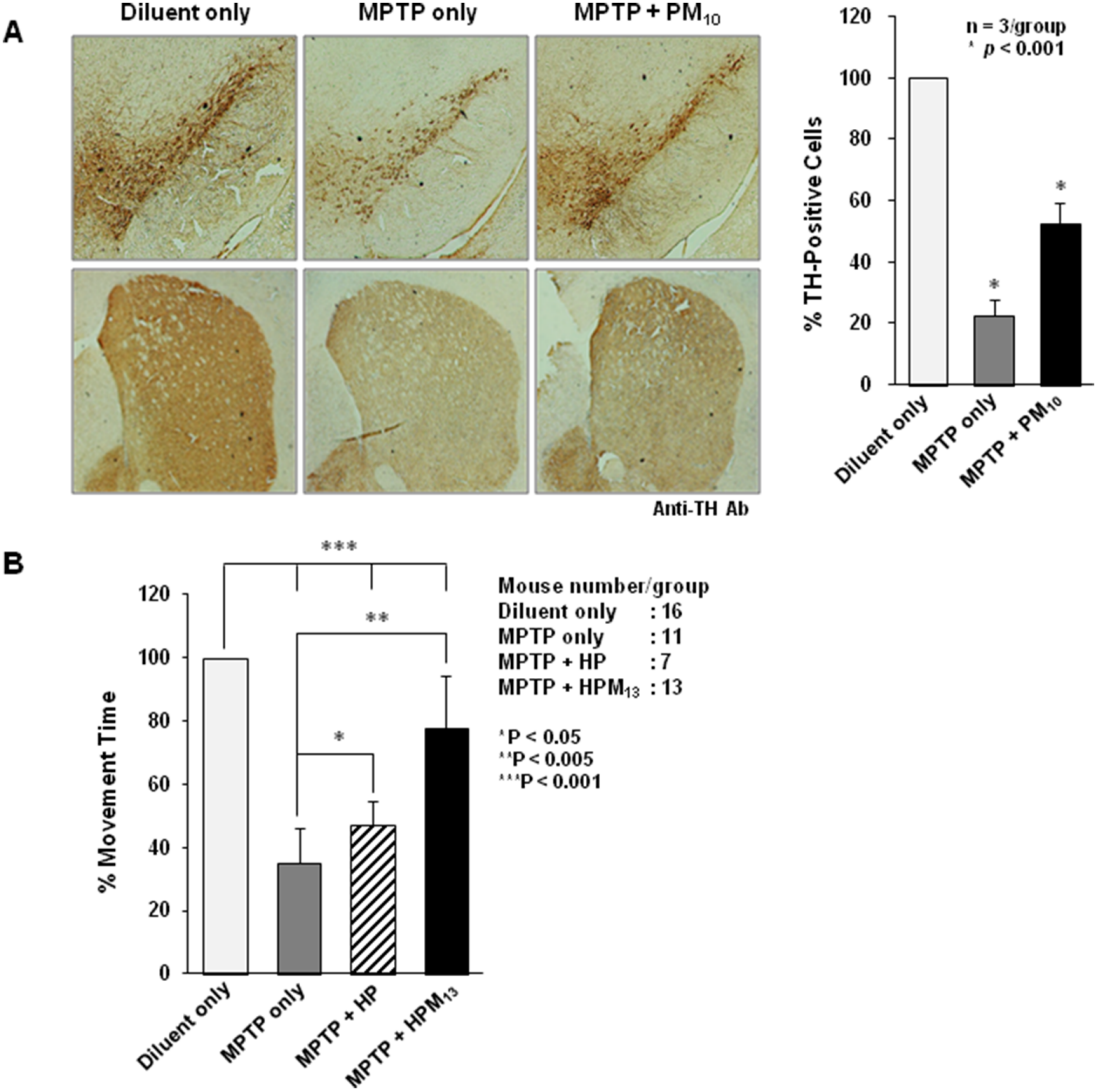
CP-Parkin reduces acute MPTP-induced neurotoxicity and preserves motor function. (**A**) PM_10_ Reduces MPTP-induced dopaminergic toxicity. MPTM-lesioned mice were treated with PM_10_ for 5 days as shown in Figure 5A (IP, 10 mg/kg) and loss/preservation of dopaminergic neurons was determined by tyrosine hydroxylase (TH) staining (left panel). (**B**) HPM_13_ preserves gross motor function of MPTM-lesioned mice. 9 hrs after the last MPTP treatment (Figure 5A) mice were treated for 3 hrs with 200 µg proteins (IP, HP or HPM_13_), and motor ability was assessed by placing the animals in a water bath and video recording subsequent movements. The percentage of time of the mice in each treatment group were engaged in 4 legged motion is presented as means ± S.D. The number of mice in each group was as follows: Diluent, 16; MPTP only, 11; MPTP+HP, 7; MPTP+HPM_13_, 13. Experimental differences between groups were assessed by ANOVA (*p<0.05, **p<0.005, ***p<0.001).

The neurotoxin, 1-methyl-4-phenyl-1,2,3,6-tetrahydropyridine (MPTP), targets dopaminergic neurons to produce clinical and pathological changes resembling Parkinson’s disease [24]. CP-Parkin proteins were tested in MPTP-treated mice for the ability to preserve dopaminergic neurons. 8-week-old C57BL/6 female mice received 3 intraperitoneal (IP) injections of 15 mg/kg MPTP at 2 hr intervals on 2 consecutive days and were treated (IP) daily over 5 days with saline or with 200 µg HP or HPM_13_ proteins (**Figure 5A**). Urine dopamine levels, measured 1 to 8 hrs after the first protein treatment (day 3), were significantly higher in HPM_13_-treated animals (**Figure 5B** **;** *p*<0.005). CP-Parkin (PM_10_) was also neuroprotective as assessed by dopamine levels 25% higher in the midbrain at day 7, including substantia nigra, of treated as compared to untreated mice (**Figure 5C**). The percent of tyrosine hydroxylase (TH)-positive cells in the striatum of MPTP-treated mice was also higher in PM_10_-treated mice (55% of unlesioned mice) as compared to 21% observed in untreated controls (**Figure 6A** **,** *p*<0.001). PM_10_ also preserved the tyrosine hydroxylase status of primary rat neurons treated in vitro with 6-hydroxydopamine (data not shown).

These experiments do not distinguish the extent to which loss of TH staining (and restoration by CP-Parkin) reflects MPTP-induced neuronal cell loss or suppression of TH expression. Most important with regard to physiological function, gross motor functions were assessed after acute MPTP-induced injury by using a swim test [25], [26]. Mice were placed in a water bath and their movements were video recorded for later blind analysis (**Figure 6B**). While the control mice swam more or less continuously (98% of the time), the lesioned animals were considerably (35%) less active. Mice treated with 6xHis tagged parkin appeared slightly more active (49%) on experimental day 3, but the effect was not statistically significant. By contrast, gross motor functions of HPM_13_-treated mice were within 25% of the normal controls (*p*<0.001) (**Figure 6B**). The comparisons are MPTP only to MPTP+HP (*); MPTP only to MPTP+HPM13 (**); Diluent to each group [MPTP only, MPTP+HP or MPTP+HPM13] (***).

## Discussion

Since parkin, PINK1 and DJ-1 are widely expressed and oxidative stress, mitochondrial poisons and misfolded proteins presumably affect many cell types, other features of the neuronal phenotype have been proposed to explain the differential vulnerability of dopaminergic (and other) neurons observed in PD [27]. These include dopamine metabolism itself [28], [29] and electrophysiologic features of neurons in the substantia nigra, characterized by slow, broad-spike, autonomous pacemaking with low Ca^2+^ buffering [30]. Both processes induce oxidative stress, placing steady-state burdens on cellular anti-oxidant defenses and mitochondrial homeostasis, which in turn are thought to enhance the vulnerability of dopaminergic neurons [27].

The present study demonstrates the effectiveness of protein-based therapy to deliver enzymatically active, cell-permeable (CP) parkin proteins as neuroprotective agents in an acute intoxication model of dopaminergic neuronal loss. CP-Parkin proteins containing MTDs displayed enhanced cellular uptake and tissue penetration as compared to proteins lacking an MTD, and they suppressed Parkinson Disease-associated phenotypes in 6-OHDA- and MPTP-treated cells and animals. In particular, CP-Parkin suppressed 6-OHDA-induced apoptosis in CATH.a and SH-SY5Y cells, stimulated dopamine expression in cultured CATH.a cells, preserved tyrosine hydroxylase positive cells in the striatum and midbrain, enhanced dopamine expression, and preserved gross motor functions (swim test) in MPTP-treated mice.

The levels of cytoprotection achieved by CP-Parkin were comparable to those reported after enforced expression of the parkin gene in neuronal cell lines and in animal models of PD [5], [7], [11]. This suggests that the activity of systemically delivered HPM_13_ and PM_10_ protein approaches theoretical limits associated with augmented parkin function in the different models examined. These results demonstrate that cytoprotective benefits of parkin do not require prolonged prior exposure but can be achieved relatively quickly, under non-steady state conditions.

Cell-permeable proteins used in the present study possessed a low intrinsic auto-ubiquitination activity characteristic of the wild type protein, and the auto-ubiquitination activity was unaltered by the addition of amino-terminal 6xHis or carboxyl-terminal MTD sequences. These results are consistent with previous studies in which parkin appeared to tolerate a variety of amino- and carboxyl-terminal sequences while maintaining normal enzymatic and regulatory functions [31]–[34]. Although, we were unable to assess potential contributions of the MTD sequence on the biological activity of CP-Parkin due to problems expressing MTD sequences in soluble form, we have observed no effects in cells or tissues treated with other cargos containing MTD sequences, e.g. EGFP (Supplementary Figure S1). Moreover, all of the biological effects attributed to CP-Parkin in cells and mice were similar to those associated with enforced *PARK2* gene expression.

Parkin ubiquitination ligase activity is intrinsically repressed due to structural features of the protein that occlude the E2 and catalytic sites [34]. Enzyme activity is regulated by mitochondrial relocalization, post-translational modifications and ligand binding to the ubiquitin-like (Ubl) domain [31]–[33], [35]–[38]. In addition, the enzyme can be constitutively activated either by deletion of amino-terminal sequences containing the Ubl and zinc-finger RING0 domains or by various missense mutations responsible for juvenile autosomal recessive PD [34]. The latter mutations destabilize the protein as a consequence of enhanced auto-ubiquitination. It will be important to test cell-permeable parkin proteins–engineered either to be constitutively active or to resist auto-ubiquitination–for enhanced cytoprotective activity.

In principle, protein-based therapeutics offers a way to control biochemical processes in living cells under non steady-state conditions and with fewer off target effects than conventional small molecule therapeutics. However, systemic protein delivery in animals has proven difficult due to poor tissue penetration and rapid clearance [39], [40]. Protein transduction exploits the ability of specific basic, amphipathic and hydrophobic protein sequences to enhance the uptake of proteins and other macromolecules by mammalian cells [39], [40]. Some success has been achieved using sequences derived from hydrophobic signal peptides to deliver biologically active peptides and proteins to a variety of tissues (including liver, lung, pancreas, lymphoid tissues and brain). The hydrophobic macromolecule transduction domains (MTDs) used in the present study were selected from a screen of more than 1,500 signal peptide sequences. Although the MTDs we developed do not have a common sequence or sequence motif they were all derived from the hydrophobic (H) regions of signal sequences (HRSSs) that also lack common sequences or motifs except their hydrophobicity and the tendency to adopt alpha-helical conformations. The wide variation in H region sequences may reflect prior evolution for proteins with membrane translocating activity and subsequent adaptation to the SRP/Sec61 machinery, which utilizes a methionine-rich signal peptide binding pocket in SRP to accommodate a wide-variety of signal peptide sequences. The persistence of H region sequence variability presumably reflects unknown cargo-specific advantages. Similarly we know that specific MTDs work better than others with specific cargos, particularly with regard to maintaining protein solubility, and have published several such examples [13], [14], [16], [18], [20]–[22], [41]–[43].

The development of MTD sequences has been largely empirical, starting with a screen for sequences able to enhance EGFP reporter protein uptake. Individual sequences were further modified to eliminate charged and polar amino acids, increase predicted α-helical content and limit the number of consecutive hydrophobic residues. These hydrophobic MTD sequences appear to penetrate the plasma membrane directly [17] after inserting into the membranes [44]. In particular, translocation of the FGF4 MTS [17] occurs after the peptide inserts into the membrane in a “bent” configuration with hydrophobic sequences adopting an α-helical conformation [44]. MTD-dependent uptake of proteins was bidirectional as evidenced by cell-to-cell transfer. Cellular uptake was also sensitive to low temperature, did not require microtubule reorganization, was not enhanced by agents that disrupt the plasma membrane, and did not utilize ATP [21]–features consistent with direct membrane penetration. Cell-permeable p18^INK4c^ traversed synthetic membranes consisting of cholesterol and phospholipid and was capable of bidirectional movement across membranes as assessed by cell-to-cell protein transfer [21]. Finally, as reviewed elsewhere [21], [43] the presence of an MTD sequence does not preclude uptake by other mechanisms, including adsorptive and fluid-phase endocytosis. As a consequence, the uptake of MTD-containing cargoes is not exclusively cytoplasmic.

Hydrophobic MTD sequences have been used to deliver biologically active peptides and proteins to variety of tissues and tumor xenografts. Striking therapeutic benefits using these MTDs have been reported using a small peptide to protect against otherwise lethal inflammatory responses [13], [14], [16], [42] and using larger cell-permeable proteins including suppressor of cytokine signaling 3 (SOCS3) to protect animals against lethal inflammation [18], the NM23 metastasis suppressor to inhibit the seeding and growth of pulmonary metastases [20], the cyclin-dependent kinase inhibitor, p18^INK4c^, to inhibit the growth of tumor xenografts [21], the RUNX3 tumor suppressor to suppress the growth of subcutaneous gastric tumor xenografts [22] and the angiogenesis inhibitor, Endostatin, to reduce the growth of human tumor xenografts [43].

In the present study we show that the hydrophobic MTD sequence is strictly required for efficient Parkin uptake by cells and for systemic delivery *in vivo*. Similarly, the widespread tissue distribution and neuroprotective activity of CP-Parkin after intra-peritoneal administration illustrates the ability of MTD-containing proteins to penetrate multiple cell and tissue barriers. By contrast, bulk entry mediated by basic protein transduction domains occurs by absorptive endocytosis and macropinocytosis. While the latter approach has suffered from poor tissue penetration and protein bioavailability [39], [40], several groups have used the Pep-1 and HIV Tat transduction sequences to deliver a variety of protein cargoes (including SAG, Frataxin, Hsp27, Cdk5, Hsp70, Metallothionein III, and DJ-1) and have reported neuronal protection in acute animal models [45]–[53] although these studies did not assess the impact of protein therapy on motor functions.

The enhanced cytoprotection and preservation of motor function achieved by CP-Parkin suggests endogenous parkin levels become rate-limiting under conditions of acute intoxication and possibly during the development of progressive PD in the elderly. In short, intrinsic parkin responses appear to be restrained, presumably at the level of basal expression, enzymatic activation and/or post-activation protein turnover initiated by auto-ubiquitination. Such functional restraint and tight biochemical regulation suggest a requirement to guard against gratuitous or compartmentally misplaced ubiquitin ligase activity. Consequently, efforts to modulate parkin activity therapeutically either by constitutively activating the enzyme or by suppressing auto-ubiquitination may be hampered by off-target effects.

Our results demonstrate that exogenously administered parkin proteins can compensate for intrinsic limitations in the parkin response to enhance cytoprotection and preserve motor function following acute intoxication and provide a therapeutic strategy to augment parkin activity and preserve motor function following acute intoxication.

## Materials and Methods

### Ethics Statement

This study was carried out in strict accordance with the recommendations in the guidelines of ‘Institutional Review Board (IRB) of the ProCell R&D Institute, ProCell Therapeutics, Inc.’. The protocol was approved by the Committee on the Ethics of Animal Experiments of the ProCell R&D Institute. All surgery was performed under sodium pentobarbital anesthesia, and all efforts were made to minimize suffering.

### Expression and Purification of Histidine-Tagged EGFP and Parkin Fusion Proteins

MTD01, MTD13, MTD10, MTD151 and MTD174 were derived from predicted signal sequences of CAC04038, NP_639877, NP_625021, NP_630126 and NP_733505, respectively. These sequences were selected from a screen of 1,500 proteins as described previously. Histidine-tagged fusion proteins containing EGFP or the full-length 53 kDa parkin protein and MTD01, MTD13, MTD101, MTD103, the FGF4 MTS (M_m_, AAVLLPVLLAAP) or a random sequence (S, SANVEPLERL) were cloned at NdeI site in pET-28a(+) (Novagen, Darmstadt, Germany) and expressed in *E. coli* BL21-CodonPlus (DE3) cells. Recombinant EGFP and parkin proteins were named using the following convention: H, E, P and M stand for the His tag; EGFP; parkin and MTD, respectively. Recombinant EGPF proteins were HSE (His-S-EGFP), HM_m_E (His-MTS-EGFP), HM_01_E (His-MTD01-EGFP), HM_10_E (His-MTD10-EGFP), HM_13_E (His-MTD13-EGFP), HM_101_E (His-MTD101-EGFP), HM_103_E (His-MTD103-EGFP), HM_151_E (His-MTD151-EGFP) and HM_174_E (His-MTD174-EGFP). Recombinant parkin proteins were HP (His-parkin), HPM_01_ (His-parkin-MTD01), HPM_13_ (His-parkin-MTD13), HPM_101_ (His-parkin-MTD101) and HPM_103_ (His-parkin-MTD103).

Histidine-tagged recombinant proteins were purified on a Qiagen Ni^2+^ affinity resin under denaturing conditions and refolded by dialysis against 0.55 M guanidine HCl, 0.44 M L-arginine, 50 mM Tris-HCl, 150 mM NaCl, 1 mM EDTA, 100 mM NDSB, 2 mM reduced glutathione, and 0.2 mM oxidized glutathione for 48 hrs at 4°C and then changed to a physiological buffer such as PBS for in vivo or PBS plus RPMI 1640 medium (1∶1) for in vitro assays. Proteins were quantified by the Bradford method, were aliquoted, and stored at −20°C. The purified proteins were judged to have minimum levels of endotoxin as assessed by the limulus amebocyte lysate (LAL) assay (Associates of Cape Cod, Inc., East Falmouth, MA).

### Expression and Purification of Histidine-Tag Free Parkin Fusion Proteins

Sequences of *E. coli* codon-optimized and histidine-tag free recombinant parkin proteins fused to MTDs were also synthesized (Genscript, Piscataway Township, NJ) and cloned at NcoI/HindIII in pUC57 vector, and then finally cloned into pET-28a(+). Histidine-tag free recombinant parkin proteins were P (parkin), PM_10_ (parkin-MTD10), PM_13_ (parkin-MTD13), PM_151_ (parkin-MTD151) and PM_174_ (parkin-MTD174).

The proteins were expressed in *E. coli* BL21-CodonPlus (DE3) cells grown to an A_600_ of 0.6 and induced for 3 hrs with 0.5 mM IPTG. Cells were harvested and disrupted by sonication (10 sec-on/20 sec-off) for 30 min in buffer A (50 mM Tris-HCl, pH 8.0, 100 mM NaCl, 0.1% Triton X-100). Inclusion body was isolated by centrifugation (5,000 rpm for 30 min at 4°C) and dissolved in buffer B (50 mM Tris-HCl, pH 10.0, 8 M urea) for overnight for denaturation. Denatured inclusion body was dialyzed against buffer C (30 mM sodium phosphate, pH 8.0, 0.02% Tween-20) for 48 hrs at 4°C for refolding. Insoluble particles were removed by centrifugation (9,000 rpm for 30 min at 4°C). Purification was conducted by ion-exchange column chromatography with AKTA Purifier FPLC system (GE HealthCare, Pittsburgh, PA). In brief, Q-Sepharose anion column was flowed with protein solution in buffer C for protein binding and washed with buffer D (30 mM sodium phosphate, pH 8.0, 30 mM NaCl) for removing the unbound proteins. Proteins were eluted with salt gradient (30 mM to 1 M NaCl) of elution buffer E (30 mM sodium phosphate, pH 8.0). All recombinant proteins were eluted at a major single peak. After purification, proteins were dialyzed against a physiological buffer.

### Analysis of Protein Uptake In Vitro and In Vivo

Recombinant proteins were conjugated to 5/6-FITC and uptake by cultured RAW 264.7 (Abelson leukemia virus transformed murine monocyte/macrophage line) and NIH3T3 cells (mouse embryo-derived fibroblasts) were assessed as described previously [21]. Briefly, RAW 264.7 cells were treated with 10 µM FITC-labeled proteins for 1 hr at 37°C, washed with cold PBS three times, and treated with proteinase K (10 µg/ml) for 20 min at 37°C to remove cell-surface bound proteins. Protein uptake was quantified by flow cytometry (FACSCalibur; BD Biosciences, Billerica, MA).

NIH3T3 cells were exposed to 10 µM FITC-proteins for 30 min and then nuclei were counter stained with 1 µg/ml propidium iodide (Sigma-Aldrich). The cells were washed three times with cold PBS and treated with proteinase K (10 µg/ml) for 20 min at 37°C to remove cell-surface bound proteins, and examined by confocal laser scanning microscopy.

Balb/c mice (6 weeks old, female) were injected intraperitoneally (IP, 300 µg/head) with FITC only or FITC-conjugated proteins. After 2 hrs, the liver, kidney, spleen, lung, heart and brain were isolated, washed with an O.C.T. compound (Sakura) and frozen on dry ice. Cryosections (15 µm) were analyzed by fluorescence microscopy.

### Immunodetection of MTD Fusion Proteins in Brain Tissue

For immunohistochemistry, 6-week-old Balb/c female mice were injected intraperitoneally with diluent (PBS) or with 200 µg His-tagged recombinant parkin proteins. After 2 hrs, the brains were isolated, fixed with 4% paraformaldehyde for 24 hrs and frozen for cryosectioning. Brain cryosections (15 µm) were immunostained with anti-6xHis tag (1∶500, Abcam) or anti-parkin (1∶500, Santa Cruz Biotechnology) monoclonal antibodies, followed by biotin-conjugated goat anti-mouse secondary antibody (Santa Cruz Biotechnology), and developed with Avidin-Biotin Complex kit (Vectastain kit, Vector Laboratories). For western blot analysis, the brains from the mice treated with proteins were isolated, homogenized in RIPA buffer (50 mM Tris-HCl, pH 7.4, 1% NP-40, 0.25% sodium deoxycholate. 0.1% SDS, 150 mM NaCl, 1 mM EDTA). The supernatant from the centrifugation (13,000 rpm for 10 min at 4°C) was analyzed by western blot that was probed with antibodies against parkin (1∶2,000) and β-actin (1∶5,000). The secondary antibody was goat anti-mouse IgG-HRP (all antibodies were from Santa Cruz Biotechnology). Separately, mice were injected intravenously (IV, 200 µg) with diluent or proteins. After 30 hrs, brain tissue lysates were analyzed with anti-parkin antibody.

For parkin proteins without 6xHis tags, 6-week-old Balb/c female mice were injected subcutaneously (SC, 200 µg×3 times, 2 hrs interval) at the left back with diluent or proteins. After 2 hrs of the last injection, the brains were isolated, fixed with 4% paraformaldehyde for 24 hrs and frozen for cryosectioning. Brain cryosections (20 µm) and lysates (100 µg/well) were immunostained with anti-MTD10 (1∶500, Peptron) polyclonal and anti-parkin (1∶2,000, Santa Cruz Biotechnology) monoclonal antibodies, respectively.

### E3 Ligase Activity of Purified Recombinant Parkin Proteins

Parkin E3 ligase activity was measured by using an auto-ubiquitination assay (Boston Biochem) conducted according to the manufacturers’ instructions. Briefly, 1 µg of purified parkin proteins were reacted with 1 µM E1, 50 µM E2, 1 mM histidine-tagged Ubiquitin and 10 mM Mg-ATP for 1 hr at 37°C, followed by western blot with anti-Ubiquitin antibody (1∶1,000, Enzo Life Science). His-NM23 (HN) was used as a negative control protein.

### Apoptosis Assays

Terminal dUTP nick-end labeling (TUNEL) assays were conducted according to the manufacturers’ instructions (Roche). Mouse dopaminergic neuronal (CATH.a) cells (Korea Cell Line Bank) were plated (3×10^3^ for low and 5×10^5^ for high confluence) and their equivalent cell density at 5% (Low) and 70% (High) confluence was confirmed by cell counting prior to the experiment. Human brain tumor (SH-SY5Y) cells (Korea Cell Line Bank) were also cultured and confirmed the equivalent cell density (50%). These cells were pre-treated with 50 µM and 100 µM 6-hydroxydopamine (6-OHDA) for 1 hr and 6 hrs respectively, followed by treatment with 2.5 µM recombinant parkin proteins for 2.5 hrs at 37°C and analyzed for changes in cell survival.

### MPTP-Induced Parkinson’s Disease Mouse Model

1-methyl-4-phenyl-1,2,3,6-tetrahydropyridine (Sigma-Aldrich, St. Louis, MO) was dissolved in 0.9% NaCl. For lesioning, 8-week-old C57BL/6 female mice received 3 intraperitoneal injections of MPTP (IP, 15 mg/kg×3 times/day, 2 hrs interval) on two consecutive days. Controls were treated with 0.9% NaCl for the same time period. We confirm that animal experiments were performed in accordance with the guidelines of the Institutional Animal Care and Use Committee.

### Measurement of Dopamine Levels in Cultured Cells, Urine And Brain Extracts

Dopamine synthesized by cultured mouse dopaminergic neuronal cells (CATH.a), present in the urine or brain or tissue extract was measured by using a commercial ELISA kit according to instructions provided by the manufacturer (GenWay, San Diego, CA). In brief, rabbit anti-dopamine antibody is added to culture supernatant, urine or tissue extract, and the immune complexes are recovered in wells coated with goat anti rabbit antibody. A second enzyme conjugated anti-dopamine antibody directed against a different epitope produces reaction products proportional to the amount of antigen as compared against a standard curve.

### Tyrosine Hydroxylase Expression

Brains from protein (PM_10_) treated mice (IP, 200 µg/day×5 consecutive days) after lesioning with MPTP were isolated. Striatum and midbrain were rapidly dissected out, the hemispheres divided and the cortex removed from the surrounding structures. The tissues were fixed with 4% paraformaldehyde for 24 hrs and cryosected (20 µm). Dopaminergic neuronal cell marker in brain - tyrosine hydroxylase (TH) was immunostained with anti-TH (1∶1,000, Millipore, Ramona, CA) monoclonal antibody, followed by biotin-conjugated goat anti-rabbit secondary antibody (1∶500, Santa Cruz Biotechnology, Santa Cruz, CA), and developed with ABC kit (Vectastain kit, Vector Laboratories, Burlingame, CA).

### Assessment of Motor Activity

Gross motor functions of MPTP-lesioned mice were assessed by using a swim test. Mice were placed in a 37°C water bath and video recorded. Unlesioned mice swam using all 4 legs 98% of the time. The percent of time of each group (MPTP only, MPTP+HP or MPTP+HPM_13_) spent swimming (4 legged) was measured and expressed as a percent of the unlesioned control.

### Statistical Analysis

All experimental data using cultured cells were expressed as means ± S.D. for at least three independent experiments. Statistical significance was evaluated using a two-tailed Student’s *t*-test or ANOVA method. Experimental differences between groups were assessed using paired Student’s *t*-tests. For animal experiments, ANOVA for comparisons between and within groups were used to determine the significance. Differences with *p*<0.05 were considered to be statistically significant.

## Supporting Information

Figure S1 **MTD-mediated protein delivery into cultured cells and animals.** MTD01, MTD13, MTD10, MTD151, and MTD174 enhance the delivery of EGFP proteins to cultured cells. **(A)** Uptake of MTD-EGFP fusion proteins by RAW264.7 cells. Cells were exposed to the indicated FITC-conjugated proteins (10 µM) for 1 hr, treated to remove cell-associated but non-internalized protein and analyzed by flow cytometry. The EGFP protein cargos contained MTDs (HME, green), a positive control (FGF4-MTS, HM_m_E, red), and a negative control (a random arbitrary sequence, HSE, blue). Other control cells were treated with 10 µM of FITC only (black thin line) or buffer alone (filled gray peak). **(B)** EGFP protein uptake by NIH3T3 cells. Cells were incubated with 10 µM FITC-conjugated recombinant MTD-EGFP proteins, an equimolar concentration of unconjugated FITC (FITC only) or vehicle (culture medium RPMI 1640, Cell only) for 30 min, then nuclei were counter stained with 1 µg/ml propidium iodide (PI), were washed and treated with proteinase K to remove non-internalized protein and visualized by fluorescence confocal laser scanning microscopy. **(C)** Systemic delivery of MTD-EGFP proteins in vivo. Cryosections (20 µm) of saline-perfused organs were prepared from mice 1 hr after intraperitoneal (IP) injection of 20 µg FITC only or 300 µg FITC-conjugated recombinant EGFP proteins fused to MTD01, MTD13, MTD10 and MTD151 and were analyzed by fluorescence microscopy.
(TIF)

Figure S2 **MTD-mediated parkin protein delivery into cells and tissues.** **(A)** Protein uptake by RAW 264.7 cells. RAW 264.7 cells were incubated with 10 µM FITC-conjugated recombinant MTD-parkin proteins, an equimolar concentration of unconjugated FITC (FITC only) or vehicle (Cell only; culture medium RPMI 1640) for 30 min were washed and treated with proteinase K to remove non-internalized protein and visualized by fluorescence confocal laser scanning microscopy. **(B)** Protein uptake by NIH3T3 cells. NIH3T3 cells were exposed to 10 µM FITC-proteins for 30 min and then nuclei were counter stained with 1 µg/ml propidium iodide (PI). The cells were treated with proteinase K (10 µg/ml) for 20 min at 37°C; washed three times with cold PBS to remove cell-surface bound proteins and examined by confocal laser scanning microscopy. **(C)** Systemic parkin protein delivery to murine tissues. Cryosections (15 µm) of saline-perfused organs were prepared from mice 2 hrs after intraperitoneal (IP) injection of 20 µg FITC (FITC only) or 300 µg FITC-labeled parkin proteins with (HPM_01_ and HPM_13_) and without (HP) the MTD sequence. Tissue distribution of the recombinant proteins (green staining) was assessed by fluorescence microscopy.
(TIF)

Table S1 **MTD sequences with enhanced protein transduction activity.** A screen of hydrophobic signal sequences identified hydrophobic sequences (Original Sequence) from the indicated protein sources with enhanced macromolecule transduction activity. These sequences were then modified (MTD Sequence) to produce MTD01, MTD13, MTD10, MTD151 and MTD174. Relative CP stands for relative cell-permeability of MTD-EGFP protein to MTS-EGFP protein determined by RAW264.7 cell uptake. MTS is derived from FGF4. MTDs were derived from the hydrophobic regions of predicted signal peptides from the indicated proteins. Helix refers to the secondary structure of the MTD sequence as appended to the cargo proteins (EGFP or parkin), as determined by the NPSA (network protein sequence analysis) program. Sequence numbers are from the Genbank and NCBI entries. Original sequence indicates the hydrophobic region of the signal sequence of the proteins. The numbers associated with the original sequence represent the amino acid numbers of the original protein.
(TIF)
