# Correction: Cell-Permeable Parkin Proteins Suppress Parkinson Disease-Associated Phenotypes in Cultured Cells and Animals

**DOI:** 10.1371/journal.pone.0116242

**Published:** 2014-12-17

**Authors:** The PLOS ONE Staff

There is an error in affiliation 4 for author Daewoong Jo. Affiliation 4 should be: Department of Surgery, Vanderbilt University School of Medicine, Nashville, Tennessee, United States of America.
