# Supplementary figures and images for: Cell-Permeable Parkin Proteins Suppress Parkinson Disease-Associated Phenotypes in Cultured Cells and Animals
Source: PLoS One. 2014 Jul 14;9(7):e102517. doi: 10.1371/journal.pone.0102517 (PMC4097392; doi:10.1371/journal.pone.0102517)

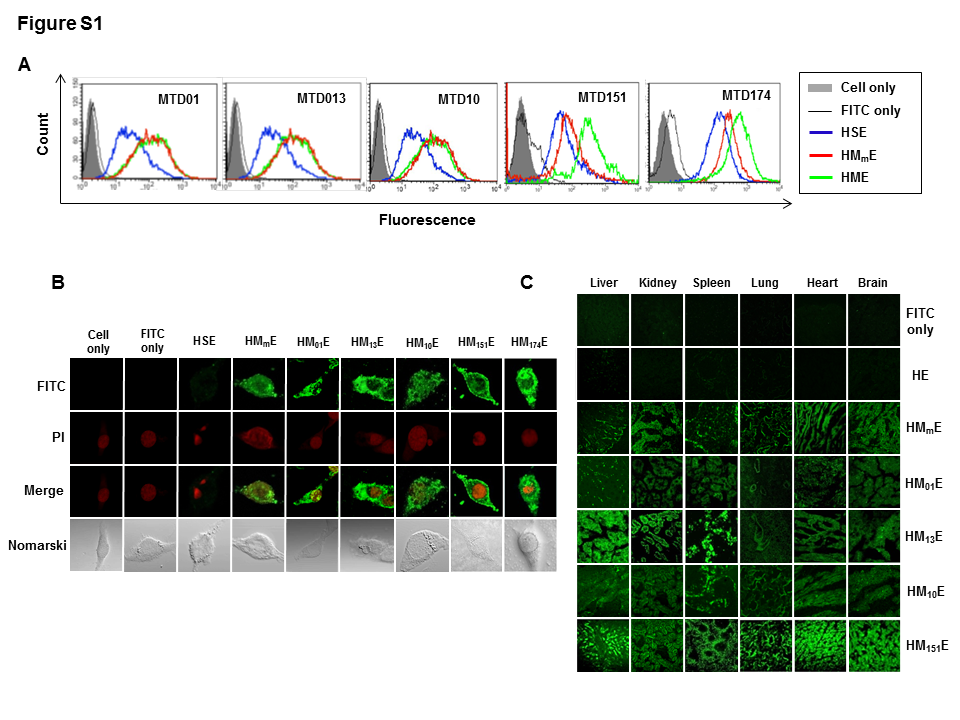

Supplement: Figure S1 — MTD-mediated protein delivery into cultured cells and animals. MTD01, MTD13, MTD10, MTD151, and MTD174 enhance the delivery of EGFP proteins to cultured cells. (A) Uptake of MTD-EGFP fusion proteins by RAW264.7 cells. Cells were exposed to the indicated FITC-conjugated proteins (10 µM) for 1 hr, treated to remove cell-associated but non-internalized protein and analyzed by flow cytometry. The EGFP protein cargos contained MTDs (HME, green), a positive control (FGF4-MTS, HMmE, red), and a negative control (a random arbitrary sequence, HSE, blue). Other control cells were treated with 10 µM of FITC only (black thin line) or buffer alone (filled gray peak). (B) EGFP protein uptake by NIH3T3 cells. Cells were incubated with 10 µM FITC-conjugated recombinant MTD-EGFP proteins, an equimolar concentration of unconjugated FITC (FITC only) or vehicle (culture medium RPMI 1640, Cell only) for 30 min, then nuclei were counter stained with 1 µg/ml propidium iodide (PI), were washed and treated with proteinase K to remove non-internalized protein and visualized by fluorescence confocal laser scanning microscopy. (C) Systemic delivery of MTD-EGFP proteins in vivo. Cryosections (20 µm) of saline-perfused organs were prepared from mice 1 hr after intraperitoneal (IP) injection of 20 µg FITC only or 300 µg FITC-conjugated recombinant EGFP proteins fused to MTD01, MTD13, MTD10 and MTD151 and were analyzed by fluorescence microscopy. (TIF) [file pone.0102517.s001.tif]

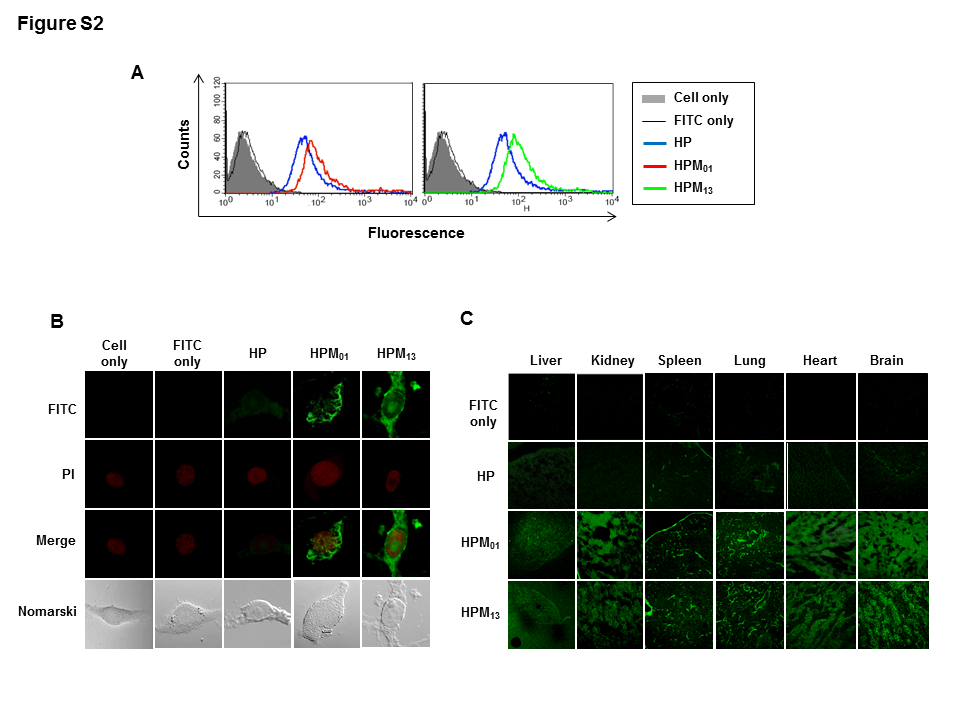

Supplement: Figure S2 — MTD-mediated parkin protein delivery into cells and tissues. (A) Protein uptake by RAW 264.7 cells. RAW 264.7 cells were incubated with 10 µM FITC-conjugated recombinant MTD-parkin proteins, an equimolar concentration of unconjugated FITC (FITC only) or vehicle (Cell only; culture medium RPMI 1640) for 30 min were washed and treated with proteinase K to remove non-internalized protein and visualized by fluorescence confocal laser scanning microscopy. (B) Protein uptake by NIH3T3 cells. NIH3T3 cells were exposed to 10 µM FITC-proteins for 30 min and then nuclei were counter stained with 1 µg/ml propidium iodide (PI). The cells were treated with proteinase K (10 µg/ml) for 20 min at 37°C; washed three times with cold PBS to remove cell-surface bound proteins and examined by confocal laser scanning microscopy. (C) Systemic parkin protein delivery to murine tissues. Cryosections (15 µm) of saline-perfused organs were prepared from mice 2 hrs after intraperitoneal (IP) injection of 20 µg FITC (FITC only) or 300 µg FITC-labeled parkin proteins with (HPM01 and HPM13) and without (HP) the MTD sequence. Tissue distribution of the recombinant proteins (green staining) was assessed by fluorescence microscopy. (TIF) [file pone.0102517.s002.tif]
